# A randomised feasibility study of EPA and Cox-2 inhibitor (Celebrex) versus EPA, Cox-2 inhibitor (Celebrex), Resistance Training followed by ingestion of essential amino acids high in leucine in NSCLC cachectic patients - ACCeRT Study

**DOI:** 10.1186/1471-2407-11-493

**Published:** 2011-11-23

**Authors:** Elaine S Rogers, Roderick D MacLeod, Joanna Stewart, Stephen P Bird, Justin WL Keogh

**Affiliations:** 1 University of Auckland, Department of General Practice and Primary Health Care, Auckland, New Zealand; 2 Auckland City Hospital, Department of Oncology, Auckland, New Zealand; 3 North Shore Hospice, Takapuna, Auckland, New Zealand; 4 University of Auckland, Department of Epidemiology and Biostatistics, Auckland, New Zealand; 5 Charles Sturt University, School of Human Movement Studies, Bathurst, Australia; 6 Bond University, Faculty of Health Sciences and Medicine, Gold Coast. Australia; 7 AUT University, Centre for Physical Activity and Nutrition Research, Auckland, New Zealand

## Abstract

**Background:**

Cancer cachexia is a syndrome of progressive weight loss. Non-small cell lung cancer patients experience a high incidence of cachexia of 61%. Research into methods to combat cancer cachexia in various tumour sites has recently progressed to the combination of agents.

The combination of the anti-cachectic agent Eicosapentaenoic acid (EPA) and the cyclo-oxygenase-2 (COX-2) inhibitor celecoxib has been tested in a small study with some benefit. The use of progressive resistance training (PRT) followed by the oral ingestion of essential amino acids (EAA), have shown to be anabolic on skeletal muscle and acceptable in older adults and other cancer groups.

The aim of this feasibility study is to evaluate whether a multi-targeted approach encompassing a resistance training and nutritional supplementation element is acceptable for lung cancer patients experiencing cancer cachexia.

**Methods/Design:**

Auckland's Cancer Cachexia evaluating Resistance Training (ACCeRT) is an open label, prospective, randomised controlled feasibility study with two parallel arms. All patients will be treated with EPA and the COX-2 inhibitor celecoxib on an outpatient basis at the study site. In the experimental group patients will participate in PRT twice a week, followed by the ingestion of essential amino acids high in leucine. A total of 21 patients are planned to be enrolled. Patients will be randomised using 1:2 ratio with 7 patients enrolled into the control arm, and 14 patients into the treatment arm. The primary endpoint is the acceptability of the above multi-targeted approach, determined by an acceptability questionnaire.

**Discussion:**

To our knowledge ACCeRT offers for the first time the opportunity to investigate the effect of stimulating the anabolic skeletal muscle pathway with the use of PRT along with EAA alongside the combination of EPA and celecoxib in this population.

**Trial registration:**

Netherlands Trial Register (NTR): ACTRN12611000870954

## Background

Cancer cachexia is a syndrome of progressive weight loss, metabolic alterations, fatigue and persistent reduction of body cell mass in response to a malignant tumour in the presence or absence of anorexia [1-3]. Cancer cachexia involves relatively similar losses of adipose (fat) and muscle tissue which differs from simple starvation or conditions such as anorexia nervosa, where the majority of weight loss is from adipose rather than muscle tissue [2,4-6].

The incidence of cachexia in cancer patients is dependent on the type and site of the tumour, and can range from 31% to 87% of all cancer patients. While low incidences are reported in Non-Hodgkin's lymphoma, breast cancer and sarcomas, rates up to 83% in pancreatic cancer patients and over 85% in patients with gastric cancer have been reported. Small-cell and Non-small-cell lung cancer (NSCLC) patients also experience a high incidence of cachexia, at 57% and 61% respectively [6-9]. It is estimated that cachexia is present in up to 80% of cancer patients at death, and the main cause of death in 20% of all cancer patients [6,9].

Cancer cachexia is associated with a deterioration of functional status and quality of life and is also associated with poor survival [6]. Cachectic patients have lower response rates to chemotherapy and shorter median survival [7]. While much of the cachectic weight loss is from adipose tissue, it has been suggested that it is the loss of muscle mass that accounts for mortality and morbidity [10]. Muscle wasting is the main cause of impaired function, leading to respiratory complications and fatigue [11].

Recently there has been a shift from the use of the cytotoxic platinum-based drug carboplatin to cisplatin in the treatment of NSCLC. Cisplatin is associated with side effects, amongst these cachexia and anorexia, thereby confounding the symptom [12].

A recent review has shown that over the last few decades a number of studies have attempted to reduce patients' cachexia. This has involved the investigation of several pharmacological agents. Unfortunately these studies demonstrated either no or limited benefit [13]. Research into methods to combat cachexia has therefore recently progressed to the combination of agents e.g. megestrol acetate and ibuprofen [14], Eicosapentaenoic Acid (EPA) and nutritional supplements, nutritional support, anti-inflammatory and anaemia support, all again with mixed results [13].

Such results would suggest that there is a need for a new approach to the management of cancer cachexia. Based on the literature for rheumatoid arthritis cachexia group [15], older adults [16], and other cancer groups [17,18] the combined use of anti-inflammatory agents, improved food intake, especially protein and the performance of progressive resistance training (PRT) anabolic exercise, to stabilize the cachectic patient would appear worthwhile investigating [13].

### Non-steroid anti-inflammatory drugs (NSAID)

The use of NSAIDs in cancer patients is not widespread due to concerns regarding cyclo-oxygenase-1 (COX-1) inhibition, and its effect on gastrointestinal mucosal lining resulting in gastrointestinal bleeding and perforation [19]. The development of selective COX-2 inhibitors has led to the possibility of their use in reducing tumour-mediated prostaglandin levels safely and could help alleviate or control cancer cachexia. The selective COX-2 inhibitor celecoxib has shown potent anti-tumour growth inhibitory and anti-tumour preventive effects in animal models [19].

The use of celecoxib has been investigated in eleven cachectic patients with head and neck and gastrointestinal cancer [20]. Patients were randomised to celecoxib 200 mg twice daily or placebo for 3 weeks. The patients on celecoxib reported good compliance and no adverse events were seen [20]. Patients receiving celecoxib showed a non-significant increase in body weight, (mean change +1.0 kg compared with placebo group mean change -1.3 kg), and a significant increase in mean change score of the Quality of life (QoL) Functional Assessment of Anorexia/Cachexia Treatment (FAACT) questionnaire compared to a decrease in the placebo group (*P *= 0.05). These results would appear to suggest that only targeting inflammatory suppression may have some benefits but will not produce a clinically significant increase in lean body mass.

Recently, Mantovani and colleagues conducted a phase II non-randomised prospective study investigating celecoxib at a dose of 300 mg per day for four months in twenty-four advanced cancer patients (mixed tumour sites) [21]. Results (see Table 1) showed a significant decrease in the pro-inflammatory cytokine tumour necrosis factor-alpha (TNF-α) levels and a significant increase in lean body mass (by bioelectrical impedance). Significant improvements were also seen in QoL European Organization for Research and Treatment of Cancer Quality of Life Questionnaire-C30 (EORTC QLQ-C30), performance status according to the Eastern Cooperative Oncology Group (ECOG ) PS scale, Glasgow prognostic score (GPS) and grip strength, along with good compliance and again no grade 3 or 4 toxicities [21].

**Table 1:** Endpoints from study investigating single agent celecoxib by Mantovani et al [21]

	Baseline	After treatment	*p *values
TNF-alpha (pg/ml)	36.8 ± 14.1	29.9 ± 12.2	0.007

LBM (Kg)	45.4 ± 6.7	45.8 ± 6.6	< 0.0001

QoL	66.3 ± 19.9	75.3 ± 10.1	0.024

ECOG PS	1.5 ± 0.67	1.2 ± 0.59	0.0023

GPS	1.3 ± 0.77	0.8 ± 0.7	0.0004

Grip strength	20. 8 ± 4.7	24.0 ± 5.5	0.004

TNF-alpha = Tumour Necrosis Factor-alpha, LBM = Lean body mass, QoL = Quality of life, ECOG PS = Eastern Cooperative Oncology Group (ECOG) Performance status, GPS = Glasgow prognostic score. Data are reported as mean ± SD.

### Eicosapentaenoic acid (EPA)

EPA is an omega-3 polyunsaturated fatty acid found in oily fish which has been shown to have anti-tumour and anti-cachexia activity in animal cachexia models [22,23]. A recent review examined the results for five hundred and eighty-seven patients across five studies and found that there was insufficient data to establish a therapeutic benefit of EPA compared to placebo or to define the optimal dose [24]. Internationally 2 g per day of EPA is used as the ideal study dose in cancer cachexia studies due to the following two studies [25,26]. Weight stability has been shown with 2 g per day in eighteen unresectable pancreatic cancer patients. While all subjects experienced progressive weight loss before treatment, three quarters became either weight-stable, or gained weight after treatment (*P *< 0.002) [26]. Furthermore, Barber et al [25] reported a significant increase in lean body mass with 2.1 g per day in twenty progressive weight losing, unresectable pancreatic cancer patients after 3 weeks (*P *= 0.024), and after 7 weeks of treatment (*P *= 0.033) [25]. Overall it has been concluded from the above review [24] along with the additional results from a large double-blind, placebo-controlled study which looked at EPA at two different dosages, that EPA on its own has only marginal effects on cachexia and that it should be used in combination with other agents [27].

### Combined therapies

A range of clinical multimodal studies have been conducted examining the combined effects of Ibuprofen and megestrol acetate [14], COX-2 (celecoxib), Medroxyprogesterone acetate and oral food supplements [28], and home total parenteral nutrition, anti-inflammatory (indomethacin) and erythropoietin therapy on cachexia [29].

### COX-2 (celecoxib) and EPA

To date only one study has examined the potential benefits of COX-2 inhibitors and EPA in reducing the effects of cachexia. This study involved twenty-two advanced stage IIIb-IV NSCLC patients randomised to 2 g per day of fish oil/placebo vs. 2 g per day of fish oil/200 mg celecoxib twice daily [30].

Results indicated that the patients in both groups showed significantly less fatigue, lower C-reactive protein (CRP) levels and increased appetite when compared to their baseline values.

When comparing both groups, results showed significantly higher increases in hand-grip scores and body weights were seen in the fish oil/celecoxib group (Table 2). Lean mass and fat mass also showed a trend of increasing in this group [30]. Total dose of EPA used in this study was 1.080 g per day, a value approximately half of previous studies which used 2 g per day [25,26].

**Table 2:** Endpoints from study investigating EPA/placebo vs. EPA/celecoxib by Cerchietti et al [30]

	Fish oil/placebo	Fish oil/celecoxib	*p *values
Grip strength	1.16 (0.3)	3.12 (0.95)*	*P *= 0.002

Body weight (Kg)	-1.4 (0.84)	1.5 (1.2)*	*P *= 0.05

LBM (Kg)	-0.6 (0.67)	0.4 (0.6)	NS

Fat Mass (Kg)	-0.6 (0.8)	1.2 (0.64)	NS

LBM = Lean body mass. Data are recorded mean with SE in parentheses.*P < 0.02 compared to their respective baseline value.

In the present study it was clinically decided that the ingestion of EPA 2 g per day along with celecoxib would be an acceptable control group medication.

### Aetiology of muscle wasting

Muscle wasting is a combination and balance of the anabolic and catabolic pathways, with decreased protein synthesis combined with increased protein breakdown [31]. It is hypothesised that the increase in protein degradation may reflect the increased activity of the ubiquitin-proteasome pathway and the lysosmal system, and that the ubiquitin-proteasome pathway has the predominant role in patients experiencing weight loss [8]. Conversely the anabolic pathway involves the activation of the P13K/Akt and mTOR pathway [32], leading to phosphorylation and activation of its downstream target proteins, the eukaryotic initiation factor 4E binding protein and p70 ribosomal S6 kinase 1[8]. The decrease in protein synthesis is hypothesised to be due to changes to the phosphorylation of these initiation factors [8].

### Progressive resistance exercise training (PRT)

While a variety of pharmacological approaches have been examined for their ability to reduce or reverse cachexia in humans, one approach that has been largely ignored is that of PRT. This is most surprising as PRT has been shown to be a potent stimulus for enhancing muscle growth and strength and mass in a variety of groups including athletes, older adults and for other cancer groups [33]. PRT may down-regulate pro-inflammatory cytokine activity and increase the phosphorylation of intramuscular amino acid signalling molecules mTOR and p70 ribosomal S6 kinase 1 [33].

PRT has been used in patients with well-controlled rheumatoid arthritis with cachexia. This phase II study reported that when PRT was performed an average 2.5 times a week for 12 weeks, significant increases in total body skeletal muscle mass occurred with an adjusted (post-test scores adjusted for pre-test scores) total lean mass of 45.0 ± 0.3 kg in the training group and by 43.8 ± 0.3 in the control group (*P*= 0.005). PRT seemed to be a safe and effective intervention with no exacerbation of the activity of the disease [15].

Aerobic exercise and PRT has become popular in the cancer community in recent years. There is now extensive literature supporting PRT as the most effective method for improving muscle function and strength, and reducing the effects of sarcopenia in older adults [34].

Physical activity has showed consistent improvements in patient rated QoL scores, by patients that are experiencing both health and disease [35]. This benefit has been investigated in patients with cancer, although mainly in the breast cancer patient population [35]. A recent New Zealand study described a positive relationship between quality of life and physical activity in prostate cancer survivors on androgen-deprivation therapy [36]. The positive relationship between physical activity levels and quality of life in these prostate cancer survivors concurs with the findings of a systematic review examining body composition, functional performance, QoL and physical benefit of exercise in prostate cancer patients [17]. Results support the benefits of exercise in improving muscular endurance, aerobic endurance and overall QoL as well as reducing fatigue in prostate cancer patients. This review also recommended that the exercise performed should include a substantial PRT component and be group-based to facilitate greater psychosocial benefits [17]. A recent systematic review examined the benefit of an exercise intervention on health related QoL and exercise capacity specifically in NSCLC patients [37]. Exercise interventions included resistance training, stretching exercises, aerobic training and education regarding exercise, under supervised or unsupervised conditions. Sixteen studies involving thirteen patient groups were assessed. The studies included two randomised controlled trials and nine case series. It was concluded that improvements were seen in patients' exercise capacity when participating in exercise pre-operatively. For patients participating in exercise post-treatment, improvements in exercise capacity was seen, but results for health-related QoL were conflicting [37]. None of the above studies looked at exercise in relation to the symptom of cachexia.

### Combined approach

In a recent review it has been shown that resistance exercise and amino acids can independently stimulate skeletal muscle synthesis and that muscle synthesis is greatly increased if amino acids, especially leucine are ingested after the resistance training exercise [38]. Studies in the older adult have confirmed that providing this nutrition after exercise increases muscle synthesis, although at a slower rate, to levels similar to younger adults [38].

Recently an open non-randomized phase II study looked at the efficacy and safety of an oral amino acid functional cluster supplementation in twenty-five cachectic cancer patients [39]. All patients had advanced cancer at mixed tumour sites. The results of this study showed a significant increase in grip strength (28.2 ± 9.5 vs. 30.4 ± 9.2, *P *< 0.0001), along with the trend of an increase in body weight (Kg) (53.1 ± 10.6 vs. 54.2 ± 11.1, *P *= 0.056). Improved levels of fatigue on the QoL multidimensional fatigue symptom inventory-short form (MFSI-SF) were seen. A decreasing score is associated with a lower level of fatigue (25 ± 8.1 vs. 22 ± 7.3, *P *= 0.181). Decreasing CRP (24.7 ± 18.1 vs. 17 ± 11.4, *P *= 0.066) and interleukin-6 (IL-6 pg/ml) levels (21.3 ± 16.4 vs. 13.7 ± 4, *P *= 0.157) were also seen. This study suggests that amino acid supplementation may be a beneficial option for the treatment of cancer cachexia, and the integration of an amino acid supplementation into a multi-dimensional approach based on diet, exercise, nutritional support and molecularly targeted drugs for the management of cancer cachexia should be the next step [39].

Such results provide further evidence that PRT is a potent anabolic stimulus and that the anabolic response to PRT can be augmented with pharmacological agents that target selected aspects of the catabolic pathways. The application of such a paradigm in cachectic cancer patients would therefore warrant investigation.

### Essential amino acids ± resistance training

Intravenous and orally administered amino acids have been investigated in a number of settings in relation to protein synthesis [40]. Exercise has been shown to also have a profound effect on both muscle protein breakdown and protein synthesis [40]. After reviewing oral composition studies [39-50], of ingestion doses between 6.7 g and 40 g, it was decided to use the amino acid composition used by the studies of Fujita et al and Dreyer et al [47,50] as reviewed in a recent systematic review (see table 3)[38]. Therefore, the ingestion dosage for the current investigation is 20 g.

**Table 3:** Essential amino acids composition

Essential amino acid	(g)	%
Histidine	1.6	8%

Isoleucine	1.6	8%

Leucine	7.0	35%

Lysine	2.4	12%

Methionine	0.6	3%

Phenylalanine	2.8	14%

Threonine	2.0	10%

Valine	2.0	10%

**Total**	**20.0 g**	**100%**

### Study rationale/purpose

The optimal treatment for cancer cachexia is the complete removal of the tumour. Unfortunately in many advanced solid tumours this is unachievable, especially in the case of NSCLC patients. The next best options are to increase nutritional intake and to counteract the weight loss, address the anorexia and reduce inflammation, along with the metabolic alterations i.e. loss of body fat and the skeletal muscle wasting [6,51].

Therefore, the current investigation aims to examine a novel treatment regimen that may alleviate and/or stabilise this weight loss. With the goal of increasing muscle anabolism with PRT and essential amino acids high in leucine post exercise, the overall aim is to stabilise the effect of muscle catabolism/anabolism to a net gain in muscle mass. The investigation shall employ a multi-targeted approach by utilizing data gained from the literature to target and decrease the pro-inflammatory cytokines by using a COX-2 inhibitor (celecoxib) and decrease the ubiquitin proteolytic pathway with a proteasome inhibitor EPA. This feasibility study is required before recruiting to a full study to determine if a multi-targeted therapy including PRT is acceptable to this study population along with gaining data around recruitment and retention and safety around this type of clinical study. Variance and intra-patient correlation of the secondary outcomes will then be used to power the main study.

## Methods/Design

### Trial organisation

Auckland's Cancer Cachexia evaluating Resistance Training (ACCeRT) is designed and coordinated by the Department of General Practice and Primary Health Care, University of Auckland, New Zealand. University of Auckland is responsible for overall trial management, regulatory affairs, statistical planning and analysis, trial registration (ACTRN12611000870954), and reporting as well as quality assurance. The trial will be performed at North Shore Hospice, Auckland, New Zealand.

### Participants

Twenty-one patients will be recruited from local hospice day centres in Auckland and from referring clinicians based at Auckland City Hospital, Auckland.

### Medication supply

EPA will be supplied by Health World Limited, celecoxib (Celebrex) by Pfizer Australia and New Zealand, and essential amino acids prepared by Musashi, Notting Hill, Australia.

### Ethics, informed consent and safety

The final protocol was approved by the Northern Y Ethics Committee, Hamilton, New Zealand (NTY/11/06/064) on the 2nd of September 2011. The clinical trial complies with the Helsinki Declaration from 2008, the Medical Association's professional code of conduct, the principles of Good clinical practice guidelines and the Federal Data Protection Act.

Written informed consent for the clinical trial ACCeRT will be obtained from each participating patient in the oral and written form before inclusion in the trial. The nature, scope and possible consequences of the trial will be explained by a physician (or Principal Investigator) in detail. The investigator will not undertake any measures for the clinical trial until valid consent has been obtained.

### Study objectives

The primary objective of this clinical trial is to evaluate whether a multi-targeted approach encompassing resistance training element is acceptable for lung cancer patients experiencing cancer cachexia. This will be assessed by the analysis of a patient-rated Likert scored questionnaire asking 10 questions on the acceptability of the above multi targeted approach, both at week12/visit 5 and end of study visit.

### Eligibility criteria

#### Inclusion criteria

**1**. Patients ≥ 18 years old

**2**. Histological confirmed non-small cell carcinoma of the lung. Histological or cytological specimens must be collected via surgical biopsy, brushing, washing or core needle aspiration of a defined lesion. Sputum cytology is not acceptable

**3**. Patients should be aware of the diagnosis of cancer

**4**. Patients able to give written informed consent obtained according to local guidelines

**5**. Karnofsky Score (KS) ≥ 60 or ECOG Performance Status 0,1, 2 or 3

**6**. Recently completed first-line platinum-based chemotherapy (minimum of 1 month post last cycle)

**7**. Life expectancy ≥ 20 weeks

**8**. Fulfils the following 'cachectic definition'

### Patient selection

NSCLC cachectic patients as determined by the following definition [52]

Q1 Has lost 5% of oedema-free body weight in the previous 3-6 months

Q2 Classification of cachexia either Mild > 5%, Moderate > 10%, Severe > 15% weight loss

Q3 If no documented weight loss, Is body mass index < 20.0 kg/m^2^

Q4 At least 3 out of the following 5

• Patient reported decreased muscle strength

• Fatigue as demonstrated in a maximum volume of oxygen (VO_2 _max) test or patient reported reduced physical activity

• Patient reported anorexia

• Low fat-free mass index (low muscle mass) by bioelectrical impedance

• Abnormal biochemistry: either CRP > 5.0 mg/L, IL-6 > 4.0 pg/ml, haemoglobin < 12.0 g/dL or hypoalbuminemia < 3.2 g/dL

#### Exclusion criteria

**1**. Concurrent use of other appetite stimulants e.g. medroxyprogestrone acetate, megestrol acetate, 4 mg daily dexamethasone or 30 mg daily prednisolone

**2**. Patients with systolic BP > 160 mmHg and/or diastolic > 90 mmHg

**3**. Pleural effusion that causes ≥ CTC grade 2 dyspnoea

**4**. Radiotherapy ≤ 2 weeks prior to randomisation. Patients must have recovered from all radiotherapy-related toxicities

**5**. Patients having central nervous system (CNS) metastases. Patients having any clinical signs of CNS metastases must have a Computerised Tomography or Magnetic resonance imagining (MRI) of the brain performed to rule out CNS metastases in order to be eligible for study participation. Patients who have had brain metastases surgically removed or irradiated with no residual disease confirmed by imaging are allowed

**6**. Patients with recent haemoptysis associated with NSCLC (> 1 teaspoon in a single episode within 4 weeks)

### Secondary objectives are

Secondary safety outcomes are: Serious Adverse Events (SAEs) and Adverse Events (AEs);

GPS; KS; Progression-free survival (PFS) at the end of the study; Overall compliance; Percentage of patients eligible from total number recruited; Response evaluation criteria in solid tumours (RECIST) data (if available).

**The following outcomes will be measured in this study, for use in planning the main study, at the following time points: **screening visit, week 1, 3, 6, 9, 12, 16 and 20 weeks after intervention commencement.

Secondary measured outcomes: Lean body mass assessed by bioelectrical impedance analysis (Tanita BC-418 Segmental Body Composition Analyzer, Tanita); QoL and fatigue assessed by the following questionnaires MFSI-SF, FAACT, and WHOQOL-BREF; Serum levels of proinflammatory 'classic cachexia cytokines' (IL-1, IL-6 and TNF-α) measured by Bio-Plex Pro assay, Bio-Rad); Hand grip strength assessed by hand grip dynamometry of the dominant hand, the average of three attempts with 1 minute rest between attempts (Jamar); Leg grip strength assessed by back/leg dynamometry of the right leg, the average of three attempts with a 1 minute rest between attempts (PE018 Back Dynamometer, Access Health).

Secondary measured outcome MRI thigh skeletal muscle values as assessed 'blinded' by University of Auckland and Auckland District Health Board MRI departments. Scans will be performed at screening visit and week 20 after intervention commencement only.

### Randomisation and standardised treatment scheme

All patients enrolled will be identifiable throughout the study. The investigator will maintain a personal list of patient numbers and patient names. Upon consent each patient will receive a unique identification number. After the patient's eligibility for randomisation has been assessed he/she will be randomly assigned to one of the two treatment arms in a 1: 2 ratio (EPA and celecoxib vs. EPA and celecoxib, PRT and EAA).

### Treatment scheme

Both study arms receive for a period of 20 weeks; 2.09 g EPA Ethical Nutrients Hi-Strength Liquid Fish Oil oral liquid (fruit punch flavour), 5.5 mls per day and 300 mg per day of celecoxib.

### Experimental group

Additionally, patients in the experimental group undergo two sessions a week for 20 weeks of a tailored PRT programme under the supervision of a trained exercise therapist. All PRT sessions will be carried out at North Shore Hospice. There will be a 5-10 minute warm up, followed by the exercise prescription, and a 5 minute cool-down. 20 g of essential amino acids high in leucine will be administered to the patients 1 hour after PRT.

All patients are invited to continue with compassionate use after the end of the study.

### Evaluation and follow-up

All patients must have appropriate laboratory analysis and MRI study conducted prior to study enrolment to meet eligibility criteria. Laboratory parameters will be obtained three weekly. The patient will be asked at each visit for any adverse event (SAE and AE) as well as concomitant medication. QoL questionnaire (MFSI-SF, FAACT and WHOQOL-BREF) will be handed out to all patients on screening visit, weeks 1, 3, 6, 9, 12, 16 and 20.

Patients can withdraw from study participation at any time. Patients will be taken off the study if unacceptable toxicity appears. Unacceptable toxicity is defined as serious side effect or irreversible grade 3 toxicity. After the individual ending of the study subjects will receive the best available medical and nutritional care. Patients will undergo MRI at the beginning and at the end of study. Patients will be tracked and followed up until death.

### Statistical consideration

The intent-to-treat population including all patients who are randomised with study medication assignment designated according to initial randomisation, regardless of whether patients receive study medication or receive a different medication from that to which they were randomised. This will be the primary population for evaluating the acceptability, and measured outcome endpoints. The safety population consisting of all patients who received at least one dose of study medication or treatment.

### Primary analysis

This will be assessed by the summary of a patient-rated Likert scored questionnaire asking 10 questions on the acceptability of the above multi targeted approach, both at week12/visit 5 and end of study visit. Medians and ranges will be estimated.

### Secondary analysis

All safety outcomes will be summarised by group. Efficacy outcomes will be analysed using general or generalised linear mixed models to obtain estimates of variances and co-variances for use in powering of a future study. Group, time and their interaction will be included as explanatory variables with a spatial covariance structure across time. Trends over time will also be examined to inform on the design of the future study (timing of measures and the most appropriate outcome measures).

## Discussion

As outlined in the background section, patients with advanced NSCLC patients experiencing cachexia often result in a shorter life-expectancy and deterioration in performance status and reduced QoL. There are data indicating that a multi-targeted approach is the way forward in this condition. This study aims to provide the information required to conduct a full study to identify a novel treatment regimen that will alleviate and/or stabilise cancer cachexia weight loss and is acceptable to patients with this condition. ACCeRT offers to our knowledge for the first time the initial step required to investigate the importance of stimulating the anabolic skeletal muscle pathway with the use of PRT followed by essential amino acids, alongside the use of this combination of the anti-cachectic agent EPA and anti-inflammatory drug celecoxib in this population.

## List of abbreviations

ACCeRT: Auckland's Cancer Cachexia evaluating Resistance Training; AEs: Adverse Events; CNS: Central nervous system; CRP: C-reactive protein; COX-1/COX-2: Cyclo-oxygenase-1/-2; ECOG: Eastern Cooperative Oncology Group; PS: Performance status; EPA: Eicosapentaenoic acid; EAA: Essential amino acids; EORTC QLQ-C30: European Organization for Research and Treatment of Cancer Quality of Life Questionnaire-C30; FAACT: Functional Assessment of Anorexia/Cachexia Treatment; GPS: Glasgow prognostic score; IL-6: Interleukin-6; KS: Karnofsky Score; MRI: Magnetic resonance imagining; MFSI-SF: Multidimensional fatigue symptom inventory-short form; NSCLC: Non-small-cell lung cancer; NSAID: Non-steroid anti-inflammatory drugs; PFS: Progression-free survival; PRT: Progressive resistance training; QoL: Quality of life; RECIST: Response evaluation criteria in solid tumours; SAEs: Serious Adverse Events; TNF-α: Tumour necrosis factor alpha.

## Competing interests

All authors declare that they have no competing interests. Disclosure: Stephen P Bird is a consultant to Musashi & PowerBar, Nestle Performance Nutrition.

## Authors' contributions

ESR conceived the study. ESR, RDM, and JWLK participated in the design of the study. JS was responsible for the statistical planning of the trial. ESR and RDM wrote the study protocol. SPB provided general oversight of the study and critical revision of the manuscript. All authors read and approved the final manuscript.

## Pre-publication history

The pre-publication history for this paper can be accessed here:

http://www.biomedcentral.com/1471-2407/11/493/prepub
